# The Association of the Copy Number Variation of the *MLLT10* Gene with Growth Traits of Chinese Cattle

**DOI:** 10.3390/ani10020250

**Published:** 2020-02-05

**Authors:** Peng Yang, Zijing Zhang, Jiawei Xu, Kaixing Qu, Shijie Lyv, Xianwei Wang, Cuicui Cai, Zhiming Li, Eryao Wang, Jianliang Xie, Baorui Ru, Zejun Xu, Chuzhao Lei, Hong Chen, Bizhi Huang, Yongzhen Huang

**Affiliations:** 1 College of Animal Science and Technology, Northwest A&F University, Yangling 712100, China; yp00787@163.com (P.Y.); xjwsci@126.com (J.X.); leichuzhao1118@126.com (C.L.); chenhong1955@126.com (H.C.); 2 Institute of Animal Husbandry and Veterinary Science, Henan Academy of Agricultural Sciences, Zhengzhou 45002, China; vincezhang163@163.com (Z.Z.); sjlyu@outlook.com (S.L.); eryaowang@outlook.com (E.W.); 3 Yunnan Academy of Grassland Animal Science, Kunming 650212, China; kaixqu@163.com; 4 Henan Provincial Animal Husbandry General Station, Zhengzhou 450008, China; sjlyu@outlook.com (X.W.); hna1920@163.com (Z.L.); brr908@163.com (B.R.); xuzejun1966@163.com (Z.X.); 5 Guyuan Branch of Ningxia Academy of Agriculture and Forestry Sciences, Guyuan 756000, China; caicuicui3c@163.com (C.C.); nmjxjl26@163.com (J.X.)

**Keywords:** copy number variations (CNVs), *MLLT10* gene, growth traits, association analysis

## Abstract

**Simple Summary:**

Copy number variation is a common genetic polymorphism, mainly represented by submicroscopic levels of deletion and duplication, caused by rearrangement of the genome. It is well-known that the copy number variation of a gene is associated with growth traits of livestock. In this study, we detected the correlation between the copy number variation of the the *MLLT10* gene and the growth traits of Chinese yellow cattle. We found that the copy number variation of the *MLLT10* gene has a significant influence on hip width, rump length, hucklebone width, and cannon bone circumference of some Chinese yellow cattle breeds. The results provide preliminary suggestions for Chinese yellow cattle breeding and new insights about the future of copy number variation (CNV) as a new promising molecular marker in animal breeding.

**Abstract:**

Copy number variation is a part of genomic structural variation and has caused widespread concern. According to the results of high-throughput screening of the *MLLT10* gene, we found that the copy number variation region of the *MLLT10* gene was correlated with bovine growth traits. We aimed to detect the *MLLT10* gene copy number variation and provide materials for the Chinese yellow cattle breed. In this study, the SPSS software was used to analyze the correlation among the copy number type of six different cattle breeds (i.e., Qinchuan, Xianan, Jiaxian, Yanbian, Sinan, Yunling) and the corresponding growth traits. The results showed the following: In Qinchuan cattle, the copy number duplication type was greater than the deletion and normal types; in Xianan cattle, the copy number duplication and normal types were less as compared with the deletion type; and in Yunling cattle, the frequency of the duplication type was dominant among the three types of copy number variants. The correlation analysis result showed that there is a significant correlation between the copy number variation (CNV) of the *MLLT10* gene and the growth traits of three cattle breeds. Furthermore, correlation analysis showed that *MLLT10* CNV had positive effects on growth traits such as hip width, rump length, hucklebone width, and cannon bone circumference (*p* < 0.05). This study provides a basis for the molecular-assisted marker breeding of cattle and contributes to the breeding of cattle.

## 1. Introduction

Chinese yellow cattle have always played an important role in Chinese agriculture, for meat, milk, and leather and cattle are indispensable in the Chinese culture. Due to the lack of mechanical production tools in early Chinese agriculture, cattle were used in Chinese agriculture for labor. However, with the development of technology, agriculture has been gradually mechanized and modernized, and the applications of cattle have gradually changed from labor serving to meat serving and milk serving. The change in local Chinese yellow cattle has been obvious. As a result of gradual studies of cattle genome, genetic inheritance and variation have been used to improve cattle production and reproduction. After long-term selection and cultivation, the meat production performance of modern beef cattle breeds is higher than that of the original varieties such as meat yield and quality [1]. Qinchuan cattle are representative species of Chinese yellow cattle, Qinchuan cattle are found in Guanzhong, Shaanxi, China. Qinchuan cattle are one of the most important local breeds in China because Qinchuan cattle have a high ratio of meat to bone, low fat content in the carcass, and high muscle content. A comparison with good foreign beef breeds such as Charolais and Angus, demonstrated that Qinchuan cattle were not inferior [2]; this experiment included two species of five excellent yellow cattle breeds in China (Qinchuan cattle and Yanbian cattle) and two other breeds (Yunling cattle and Xianan cattle).

In recent years, studies have found that copy number variation has an important influence on the economic traits of various cattle, and therefore variation of this structural variation causes widespread concern, which is different from single nucleotide polymorphisms. As one form of the genome structural variations, copy number variation includes duplication, deletion, and normal, meanwhile, copy number variation can be stably detected for structural variants ranging in size from 1 kb to 3 Mb [3]. The term single nucleotide polymorphism (SNP), in the 1990s, first appeared in the journal of human molecular genetics. In-depth research, has found that single nucleotide polymorphisms (SNPs) are widely present in the genomes of various organisms, but some studies have found that copy number variation (CNV) contains more total nucleotides, and appears more frequently than SNP [4]. Structural variability in the genome directly or indirectly regulates gene dosage through various mechanisms and copy number variation is widely found in mammalian genomes as a form of structural variation which has a certain impact on phenotypic variation [5] and disease [6]. CNV research has been extended to all types of livestock, including chicken [7,8], pig [9,10], goat [11], and sheep [12]. At the same time, more and more people are paying attention to the link between CNV and cattle health and production performance [13,14].

The *MLLT10* gene is located at 10p13 and most research is currently on the study of leukemia and meningioma. Acute megakaryoblastic leukemia is rare. In children and adults with acute myeloid leukemia, the *MLL* gene is fused to dozens of chaperone genes. Among these genes, the activity of chaperone 10p12 is frequent, and *MLLT10* plays an important role as a transcriptional activator in this process [15,16]. 

At present, there are few reports on the correlations among the *MLLT10* gene and bovine growth traits. The studies have only found the copy number variation region of the *MLLT10* gene by sequencing. Therefore, the purpose of this study was to investigate the possible regulation of the *MLLT10* gene on growth traits, and to provide a basis for the *MLLT10* gene copy number variation as a molecular-assisted marker for beef cattle breeding.

## 2. Materials and Methods

### 2.1. Animal Selection and Measurement of Body Size Data

All experiments were approved by the Review Committee for the Use of Animal Subjects of Northwest A&F University. In order to study the distribution of the *MLLT10* gene copy number variation in different breeds of cattle, we selected the following six Chinese beef cattle breeds: Qinchuan cattle (QC) (Qinchuan beef improvement center, Fufeng, Shaanxi), Jiaxian cattle (JX) (Jia, Henan), Xianan cattle (XN) (Biyang, Zhumadian, Henan), Yunling cattle (YL) (Xiaoshao, Yunnan), Yanbian cattle (YB) (Yanbian, Jilin), and Sinan cattle (SN) (Tongren, Guizhou). All of the above cattle were weaning-to-slaughtered, corn silage freely-fed adult female cattle. We chose a total of 515 cattle (QC = 112, JX = 85, XN = 158, YL = 96, SN = 32, and YB = 32; all samples were female and ranged in age from 1.5 to 3 years old) and studied the distribution of copy number variation types in these Chinese cattle breeds. All samples were randomly selected for use in the experiments. We used the same breed of cattle from the same cattle breeding farm to reduce error. Body size data such as height, cross height, chest circumference, chest width, chest depth, ankle length, sciatic width, waist width, weight, and tube circumference were measured for association analysis.

### 2.2. Sample Collected, Genomic DNA Extraction

We selected QC, JX, X, YL, YB, and SN cattle as sample source varieties. We ensured that the adult cattle that provided the blood samples were in good health and consistent physical condition. The extracted blood samples were frozen in liquid nitrogen and stored in a refrigerator at −80 °C until use. Extracting blood samples and extracting genomic DNA from the blood samples were performed according to the phenol-chloroform method described by Köchl S et al. [17]. 

### 2.3. Primer Design and Amplification Detection

**Figure 1:**
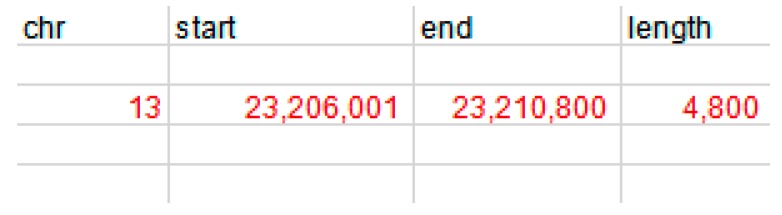
The region of the *MTTL10* gene copy number variation (CNV) in bovine genome. The copy number variation region of the *MLLT10* gene is located at Chr13:23, 206,001 to 23,210,800 bp of the *MLLT10* gene, a total of 4800 bp.

**Figure 2:**
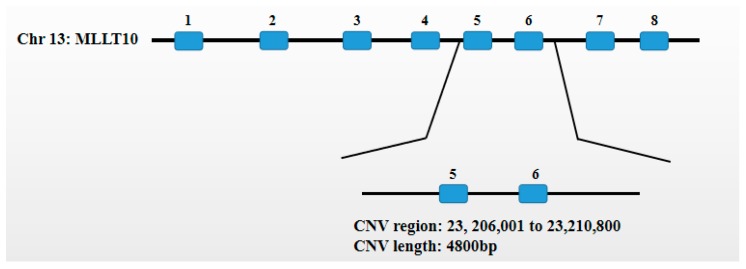
Blue boxes represent exons. The copy number variation region of the *MLLT10* gene comprised the fifth exon and the sixth exon.

We found that the *MLLT10* gene was associated with the development of adipose tissue and according to existing comparative genomic hybridization(CGH) analysis results, we detected the CNV of the *MLLT10* gene of the Chinese cattle genome. The copy number variation region of the *MLLT10* gene (NC_037328.1) is located at Chr13:23, 206,001 to 23,210,800 bp of the *MLLT10* gene, a total of 4800 bp (Figure 1). According to the method provided by National Center for Biotechnology Information (NCBI), we changed the genomic sequence to the present version to find the homologous position, and found this CNV region comprised the fifth exon and the sixth exon (Figure 2). We designed the distinct primer in the autocephalous region of the *MLLT10* gene, and simultaneously, used primer v5.0 software (PREMIER Bio soft International, California, USA) the design the primer of *BTF3*. Primer information and expression profile primer information of the *MLLT10* gene are shown in Table 1. The primer pair of the CNV region of the *MLLT10* gene is shown in Figure 1. 

### 2.4. Detection of the MLLT10 Gene Copy Number

In this research, genomic quantitative PCR (qPCR) was used to inspect the copy number variation of the *MLLT10* gene. Basic transcription factor 3 (BTF3) was chosen to be the internal reference gene due to the fact that it is highly conservative, and neither CNV nor segmental duplication appear in BTF3 [18]. According to the assumption that there were two copies of the DNA segment in the calibrator animals, we confirmed the copy number of the *MLLT10* gene. We adopted genomic qPCR experiments by using SYBR^®^ Green (Genstar, Beijing, Chinal) in three repetitions per group. The reaction system was 12.5 microliters, including 6.25 microliters of SYBR^®^ Premix Ex TaqTM II (TaKaRa, Dalian, China), 4.25 microliters of ultrapure water, primer pair consisting of 0.5 microliters of forward primer and reverse primers, and 1 microliter of DNA (10 ng). The thermal cycling conditions were a total of 39 cycles, each cycle included: 95 °C for 10 min followed by 39 cycles at 95 °C for 15 s, 60 °C for 60 s, and 65 °C for 5 s.

### 2.5. Data Analysis Processing

Cycle threshold (Ct) means the number of cycles experienced when the fluorescent signal in each reaction tube reaches a set threshold. The cycle threshold is an important parameter in the quantitative real-time polymerase chain reaction, which is used to draw a standard curve to detect the initial copy number of the template in the sample to be tested. In this study, SPSS software was used to analyze the correlation between the body size data of Qinchuan cattle, Xianan cattle, Jixian cattle, and Yunling cattle and the corresponding individual *MLLT10* gene copy number variation types by one-way analysis of variance, In the data processing, according to the different factors affecting the body size traits, considering age and genetic effects, the fixed model was used for analysis, and simplified according to the actual situation. The complete model is as follows:

Y_ijk_ = μ + A + G_j_ + E_ijk_ (1)

where Y_ijk_ is an individual phenotypic record, μ is the population mean, A_i_ is the age effect, Gj is the copy number variation type of each point, and E_ijk_ is a random error. Gene expression abundance is derived from the 2^−ΔΔCt^ method, ΔCt = Ct (target gene) – Ct (reference genes). There are three types of copy number variations, i.e., duplication (>0.5), deletion (<−0.5), and normal (<±0.5).

## 3. Results

### 3.1. Expression Profile of the MLLT10 Gene

**Figure 3:**
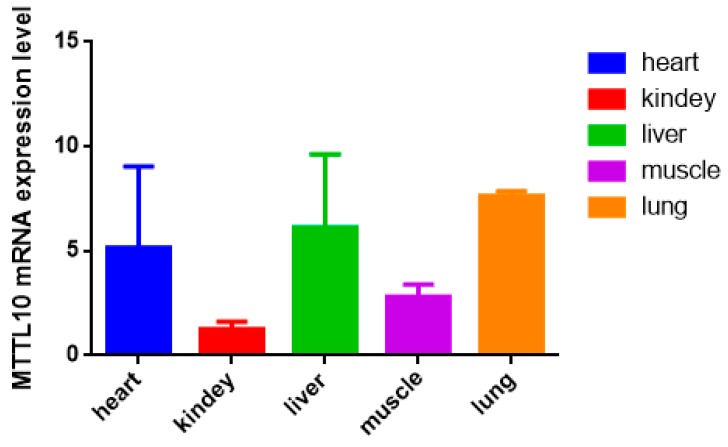
The expression test in different tissues of *MTTL10* gene in Xianan (XN) cattle.

The gene affects the growth and differentiation of a variety of tissues such as fat, heart, and muscle. Here, we reveal the expression profiles of the *MLLT10* gene in five different tissues of XN cattle (Figure 3). The results of the data analysis show that the overall expression level of the *MLLT10* gene in heart, lung, kidney, liver, and muscle was not high, and the expression in kidney and muscle was lower than that in heart, liver, and lung.

### 3.2. Distribution of the MLLT10 Gene Copy Number in the Experimental Sample Group

**Figure 4:**
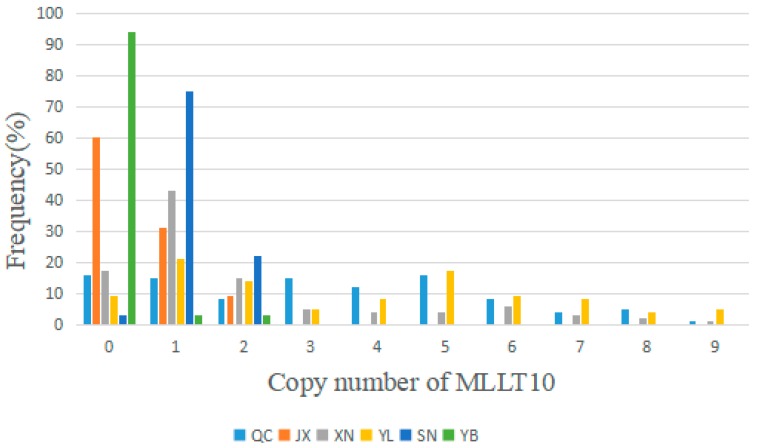
The frequency of CNV in six Chinese cattle breeds. Distribution of different copy number variants of the *MLLT10* gene in 6 different Chinese yellow cattle breeds.

As described above, in order to determine the distribution of the *MLLT10* gene copy number in some Chinese yellow cattle breeds, we chose six cattle breeds as sample species, including QC, JX, XN, YL, SN, and YB cattle. According to the log2^2−ΔΔCt^ values, we divided the CNV types into three classes, i.e., deletion (<0.5), normal (<±0.5) and duplication (>0.5), then, we converted them to deletion (0 to 2), normal (2), and duplication (>2). As shown in Figure 4, the type compositions of *MLLT10* CNV showed that the number number duplication type on the *MLLT1*0 gene CNV was more than deletion and normal in the QC and YL cattle and the copy number deletion type was more than normal and duplication types, especially in the YB and SN cattle. In Figure 4, the frequency of different CNV types in six cattle breeds is represented. As the histogram shows, copy number variation concentrate in zero and one, which suggest that the deletion type was more frequent than normal and duplication types in JX, XN SN, and YB cattle. Furthermore, we confirm that copy number deletion type was the main variation in JX, SN, XN, and YB cattle. The copy number variation type in QC cattle is more abundant than that of the other cattle breeds.

### 3.3. Correlation Analysis in the MLLT10 Gene CNVs and Growth Traits of Different Cattle Breeds

In recent years, there have been many experiments confirming that there is a clear correlation between the copy number variation of genes and the growth traits of animals [19,20]. In this research, we used SPSS software and one-way ANOVA to analyze the association among the growth traits of four bovine breeds (QC, XN, JX, YL) and the CNV of the *MLLT10* gene. After standardizing the copy number variation types of the above four cattle breeds, correlation analysis was performed separately. Meanwhile, there are three copy number types, namely deletion (0 to 2), normal (2), and duplication (>2). In QC and JX cattle, the CNV of the *MLLT10* gene lacks a significant correlation to growth traits, for which, we only presented the distribution of the CNV of the *MLLT10* gene in QC and JX cattle groups. Regarding the type of *MLLT10* CNV in XN cattle (Table 2), the duplication type in Xianan cattle individual cannon bone circumference has better performance than the deletion and normal types (*p* < 0.05). Regarding the type of *MLLT10* CNV in YL cattle (Table 2), the normal type is better than the deletion and duplication type in rump length (*p* < 0.05), conversely. The normal type of copy number variation in YL cattle is inferior to the deletion and duplication type in hucklebone width. All of results suggest that the CNV of *MLLT10* is significantly associated with cattle growth traits.

## 4. Discussion

Genomic structural variation is composed of copy number variation and single nucleotide polymorphism, which have been recognized as sources of genetic variation, but the specific function of CNV is still unclear. Recent studies have found that CNV is not only widely present in animals but also related with some traits [21]. It is notable that each copy number variable region genome encompassed more nucleotides than SNPs, suggesting that CNV is important in genetic diversity and biodiversity [22]. There are many methods to identify copy number variation region which include: CNV-seq, CNVnator, SegSeq, event-wise testing (EWT) and others. We chose qPCR as the method to detect CNV of the *MLLT10* gene. The qPCR method has the advantages of being fast, convenient, and accurate and is universally used to detect CNVs. In this study, we selected 2 × 2^−^^△△Ct^ as the analyze method and used the *BTF*3 gene as an internal reference gene. We examined the mRNA expression profile of the *MLLT10* gene in different tissues of yellow cattle and correlated the CNV type with the growth traits of yellow cattle (QC, XN, YL, and JX cattle).

By identifying the type of *MLLT10* gene’s CNV in six cattle breeds, as well as the correlation analysis with the growth traits of four yellow cattle breeds, we found that the *MLLT10* gene copy number variation type is different in different yellow cattle breeds. In terms of copy number variation type, in QC cattle, there are no significant correlations among the copy number variation of the *MLLT10* gene and growth traits. In JX cattle, the number of deletion type is much higher than others; in XN cattle, the number of deletion type is higher than others evidently; in YL cattle, the frequency of normal type is less than the duplication and deletion type. Lastly, in YB and SN cattle, the deletion type is obviously dominant, but because the number of YB and SN cattle samples was not enough to support highly reliable analysis results, we only identified the type of copy number variation, in YB cattle groups and SN cattle groups. The deletion type occupy the vast majority of three kinds of copy number variations. The distribution of the CNVs of the *MLLT10* gene in different breeds of cattle is different and rich in performance and can contain excellent breeding resources. As excellent Chinese yellow cattle breeds, performance about the CNV of the MLLT10 gene in QC cattle is absolutely distinct from YB cattle and SN cattle. The YL cattle is a Chinese hybrid breed and the distribution of the CNV of the MLLT10 in YL cattle is the same as the QC cattle, and the body types of the QC cattle and YL cattle are greater than the YB cattle and the SN cattle. The XN cattle is also a Chinese hybrid breed, whose female inheritance is from Henan Nanyang cattle, and the JX cattle and XN cattle possessed analogous tendency for the CNV of the MLLT10 gene. All of these could by caused by blood relation and the environment. This suggests that we need to study the genetic relationship between these numerous cattle breeds to further study the reasons for the differences in growth traits caused by different copy number types.

At present, most of research about the *MLLT10* gene is about disease, and it has been verified that the *MLLT10* gene variation at 10p12.31 near *MLLT10* influences meningioma risk [23]. Research has shown that the strongest evidence of association is found with the SNP rs7919823 at chromosome 10p12.21 near the *MLLT10* locus and this SNP, for each abdominal fat phenotype analyzed, showed stronger evidence of association in women than in men [24]. In addition, it has been speculated that the *MLLT10* gene has an effect on the fat development of cattle. According to the results of this study, the copy number variation of the *MLLT10* gene obviously influenced the growth traits such as cannon bone circumference, rump length, and hucklebone width of two (XN, YL) cattle breeds. Wang et al. [25] found that the *MLLT10* gene correlated with male Duroc pigs 100 KG (D100) and feed conversion ratio (FCR), however, the function of *MLLT10* gene in cattle lacks detailed explanation, and therefore suggests that the function of the *MLLT10* gene in cattle is a relatively unknown area and still needs extensive study.

We detected the expression of *MLLT10* mRNA in five different tissues of Xianan cattle and found that the expression in heart, liver, and lung is higher than that in muscle tissue and kidney. Combined with the previously mentioned finding that *MLLT10* can be related to fat development, and the fact that the expression of *MLLT10* gene in the liver is high in the experiment and the liver is the main organ of fat metabolism, it suggests that this gene could be related to fat metabolism. The differences in biological events represented by the *MLLT10* gene in different tissues affects adipose tissue growth, and it can be used for future bovine molecular marker breeding. These changes in the biological function of *MLLT10* will affect fat growth and development, and it can be used for future bovine molecular marker breeding.

## 5. Conclusions

In summary, we examined the copy number variation of the *MLLT10* gene in different Chinese cattle breeds and clearly demonstrated that this degree of variation affects the growth of cattle in a certain way, and this conclusion was confirmed by correlation analysis. In XN cattle, the CNV of the *MLLT10* gene has an obvious influence on cannon bone circumference; in YL cattle, the CNV of the *MLLT10* gene is associated with rump length and hucklebone width; in JX cattle, the CNV of the *MLLT10* gene is not associated with growth traits.

We, then, tested for expression of the *MLLT10* gene mRNA levels in Chinese bovine tissues. Growth traits are influenced by the CNV of the *MLLT10* gene in three breeds of Chinese yellow cattle. According to the above results, we revealed that the *MLLT10* gene plays an important role in the process of cattle body tissue growth. Our research demonstrates the effects of *MLLT10* CNV in some breeds of Chinese yellow cattle, for the first time, in order to provide evidence for the CNV of the *MLLT10* gene as a potential factor for bovine growth traits.

## Figures and Tables

**Table 1:** Primer information in CNV and expression test.

Primer		Sequence (5′–3′)	Product Length
*MLLT10*-CNV	Forward primer	TCCAAGGACAAGAACCCTGC	114 bp
	Reverse primer	GCTCCTCTTAGGCCCTTGTC	
BTF3	Forward primer	AACCAGGAGAAACTCGCCAA	166 bp
	Reverse primer	TTCGGTGAAATGCCCTCTCG	
*MLLT10* (mRNA)	Forward primer	GGAAGTCTCTGCGCACACTA	175 bp
	Reverse primer	TGAAAAACTGCCTTGCTGGA	
β-actin (mRNA)	Forward primer	GTCATCACCATCGGCAATGAG	84 bp
	Reverse primer	AATGCCGCAGGATTCCATG	

**Table 2:** Correlation analysis between copy number variation of the *MLLT10* gene and Chinese yellow cattle growth traits.

Chinese Yellow Cattle Breeds	Growth Traits	CNV Types (Average Value ± Standard Error)			*p* Value
		Deletion (n = 26)	Normal (n = 12)	Duplication (n = 56)	
YL	Rump Length (cm)	51.73 ± 4.285 ^a^	52.67 ± 3.367 ^a^	49.98 ± 3.952 ^b^	0.045 *
	Hucklebone Width (cm)	22.85 ± 2.572 ^a^	20.58 ± 2.712 ^b^	22.79 ± 2.708 ^a^	0.031 *
XN		Deletion (n = 70)	Normal (n = 22)	Duplication (n = 13)	
	Cannon Bone Circumference (cm)	19.19 ± 1.477 ^a^	19.23 ± 1.343 ^a^	20.38 ± 1.261 ^b^	0.022 *

Notes: Values with different superscripts (^a, b^) and * within the same row differ significantly at *p* < 0.05.

## References

[1] Paul S., Jung-Woo C., Urmila B., Jennifer M., Yan M., Xiaoping L., Stephen M. (2011). Whole genome resequencing of Black Angus and Holstein cattle for SNP and CNV discovery. BMC Genom..

[2] Yujia S., Xianyong L., Chuzhao L., Chunlei Z., Hong C. (2015). Haplotype combination of the bovine CFL2 gene sequence variants and association with growth traits in Qinchuan cattle. Gene.

[3] Feuk L., Carson A.R., Scherer S.W. (2006). Structural variation in the human genome. Nat. Rev. Genet..

[4] Stankiewicz P., Lupski J.R. (2010). Structural variation in the human genome and its role in disease. Ann. Rev. Med..

[5] Fadista J., Thomsen B., Holm L.E., Bendixen C. (2010). Copy number variation in the bovine genome. BMC Genom..

[6] Estivill X., Armengol L. (2007). Copy number variants and common disorders: Filling the gaps and exploring complexity in genome-wide association studies. PLoS Genet..

[7] Wang X., Nahashon S., Feaster T.K., Bohannon-Stewart A., Adefope N. (2010). An initial map of chromosomal segmental copy number variations in the chicken. BMC Genom..

[8] Richard P.M.A.C., Mark S.F., Tomas W.F., Shurnevia S., Hans H.C., Pete K., Richard R., Martien A.M. (2013). Large scale variation in DNA copy number in chicken breeds. BMC Genom..

[9] Fadista J., Nygaard M., Holm L.E., Thomsen B., Bendixen C. (2008). A snapshot of CNVs in the pig genome. PLoS ONE.

[10] Li Y., Mei S., Zhang X., Peng X., Liu G., Tao H., Wu H.Y., Jiang S., Xiong Y., Li F. (2012). Identification of genome-wide copy number variations among diverse pig breeds by array CGH. BMC Genom..

[11] Fontanesi L., Beretti F., Riggio V., Gómez G.E., Dall’Olio S., Davoli R., Russo V., Portolano B. (2009). Copy number variation and missense mutations of the agouti signaling protein (ASIP) gene in goat breeds with different coat colors. Cytogenet. Genome Res..

[12] Fontanesi L., Beretti F., Martelli P.L., Colombo M., Dall’Olio S., Occidente M., Portolano B., Casadio R., Matassino D., Russo V. (2011). A first comparative map of copy number variations in the sheep genome. Genomics.

[13] George E., Derek M. (2012). Bickhart. Copy number variation in the cattle genome. Funct. Integr. Genom..

[14] Da Silva J.M., Giachetto P.F., da Silva L.O., Cintra L.C., Paiva S.R., Yamagishi M.E.B., Caetano A.R. (2016). Genome-wide copy number variation (CNV) detection in Nelore cattle reveals highly frequent variants in genome regions harboring QTLs affecting production traits. BMC Genom..

[15] Morerio C., Rapella A., Tassano E., Rosanda C., Panarello C. (2005). MLL–MLLT10 fusion gene in pediatric acute megakaryoblastic leukemia. Leukemia Res..

[16] Schoch C., Schnittger S., Klaus M., Kern W., Hiddemann W., Haferlach T. (2003). AML with 11q23/MLL abnormalities as defined by the WHO classification: Incidence, partner chromosomes, FAB subtype, age distribution, and prognostic impact in an unselected series of 1897 cytogenetically analyzed AML cases. Blood.

[17] Köchl S., Niederstätter H., Parson W. (2005). DNA extraction and quantitation of forensic samples using the phenol-chloroform method and real-time. Forensic DNA Typing Protocols.

[18] Bickhart D.M., Hou Y., Schroeder S.G., Alkan C., Cardone M.F., Matukumalli L.K., Song J., Schnabel R.D., Ventura M., Taylor J.F. (2012). Copy number variation of individual cattle genomes using next-generation sequencing. Genome Res..

[19] Xu Y., Shi T., Cai H., Zhou Y., Lan X., Zhang C., Lei C., Qi X., Chen H. (2014). Associations of MYH3 gene copy number variations with transcriptional expression and growth traits in Chinese cattle. Gene.

[20] Chen C., Qiao R., Wei R., Guo Y., Ai H., Ma J., Ren J., Huang L. (2012). A comprehensive survey of copy number variation in 18 diverse pig populations and identification of candidate copy number variable genes associated with complex traits. BMC Genom..

[21] Sebat J., Lakshmi B., Troge J., Chi M., Navin N., Lucito R., Healy J., Hicks J., Ye K., Reiner A. (2004). Large-scale copy number polymorphism in the human genome. Science.

[22] Richard R., Shumpei I., Karen R.F., Lars F., George H.P., Daniel A., Heike F., Michael H.S., Andrew R.C., Wenwei C. (2006). Global variation in copy number in the human genome. Nature.

[23] Dobbins S.E., Broderick P., Melin B., Feychting M., Johansen C., Andersson U., Brännström T., Schramm J., Olver B., Lloyd A. (2011). Common variation at 10p12. 31 near *MLLT10* influences meningioma risk. Nat. Genet..

[24] Sung Y.J., Pérusse L., Sarzynski M.A. (2016). Genome-wide association studies suggest sex-specific loci associated with abdominal and visceral fat. Int. J. Obesity.

[25] Kejun W., Dewu L., Jules H.-S., Jie C., Chengkun L., Zhenfang W., Meiying F., Ning L. (2015). Genome wide association analysis reveals new production trait genes in a male Duroc population. PLoS ONE.

